# Long lasting anxiety following early life stress is dependent on glucocorticoid signaling in zebrafish

**DOI:** 10.1038/s41598-022-16257-5

**Published:** 2022-07-27

**Authors:** Jacqueline S. R. Chin, Tram-Anh N. Phan, Lydia T. Albert, Alex C. Keene, Erik R. Duboué

**Affiliations:** 1 Jupiter Life Science Initiative, Florida Atlantic University, 5353 Parkside Drive, Jupiter, FL 33407 USA; 2 College of Arts and Sciences, Department of Biology, Texas A&M University, 3258 TAMU, College Station, TX 77843 USA

**Keywords:** Stress and resilience, Developmental biology, Neuroscience

## Abstract

Chronic adversity in early childhood is associated with increased anxiety and a propensity for substance abuse later in adulthood, yet the effects of early life stress (ELS) on brain development remain poorly understood. The zebrafish, *Danio rerio*, is a powerful model for studying neurodevelopment and stress. Here, we describe a zebrafish model of ELS and identify a role for glucocorticoid signaling during a critical window in development that leads to long-term changes in brain function. Larval fish subjected to chronic stress in early development exhibited increased anxiety-like behavior and elevated glucocorticoid levels later in life. Increased stress-like behavior was only observed when fish were subjected to ELS within a precise time window in early development, revealing a temporal critical window of sensitivity. Moreover, enhanced anxiety-like behavior only emerges after two months post-ELS, revealing a developmentally specified delay in the effects of ELS. ELS leads to increased levels of baseline cortisol, and resulted in a dysregulation of cortisol receptors’ mRNA expression, suggesting long-term effects on cortisol signaling. Together, these findings reveal a ‘critical window’ for ELS to affect developmental reprogramming of the glucocorticoid receptor pathway, resulting in chronic elevated stress.

## Introduction

Development and function of the vertebrate brain are influenced by environmental cues and experience in early life^1,2^, yet our understanding of how such environmental cues in specific developmental time windows influences brain development is limited. Chronic stress in early life has robust and long-lasting effects on health and physiology that persist into adulthood^3–5^, yet how early life stress (ELS) impacts the developing brain to cause aberrant behaviors in later life remains poorly understood. In mammals, ELS has been shown to cause epigenetic and expression differences in several stress-related genes^6–10^, and can lead to impaired neuronal proliferation and morphology^11–16^. These changes impact the function of several brain regions including the hippocampus, amygdala, and hypothalamus, suggesting ELS impacts brain function throughout development^7,9,17,18^. Though its effects are well accepted, a mechanistic understanding of how ELS impairs brain function requires identifying the neuronal changes induced by specific stressors and assessing their impact brain-wide across development.

The zebrafish, *Danio rerio,* is a powerful model for studying how brain development is impacted by stress^19–21^. Both behavioral and physiological responses to stress are highly conserved among fish and mammals^22,23^. Behaviorally, both adult and larval zebrafish exhibit stereotyped responses following presentation of an aversive or unfamiliar cue including prolonged freezing, reduced exploration, thigmotaxis, and erratic swimming^24–26^. Moreover, several assays have been described and standardized for examining stress in both adults and larvae^25,27–29^. In addition to behavioral reactions to aversive stimuli, fish also display robust physiological responses to stress. Following the presentation of a stressful stimulus, the hypothalamic-pituitary-interrenal (HPI) axis, analogous to the mammalian hypothalamic–pituitary–adrenal (HPA) axis, induces a cascade of events that culminate in the production and release of cortisol^22,30–32^. Like mammals, cortisol then binds to glucocorticoid (GR) and mineralocorticoid receptors (MR) in the brain^30^. Manipulation of glucocorticoids in early development has also been shown to alter hatching times and swimming properties in the zebrafish model^33^. Combining this fish model of stress with approaches to examine brain development and function has the potential to unravel the mechanistic basis for the effects of ELS on brain development and function.

In this study we induce ELS by applying unpredictable mild electric stimuli at different developmental time points to zebrafish larvae, and measure stress behavior later in juvenile stages. Similar to mammals, ELS in zebrafish at early time points, but not late stages, leads to increased stress behaviors and elevated cortisol levels in later life. Pharmacological analysis of neuroendocrine signaling suggests that ELS disrupts development of cortisol receptors in the brain. Together, these data demonstrate that the effects of ELS are conserved from teleosts to mammals, and point to the zebrafish as a powerful genetic system for studying how ELS impacts brain development, physiology, and function.

## Results

### Zebrafish subjected to ELS have increased anxiety-like behaviors as juveniles

**Figure 1:**
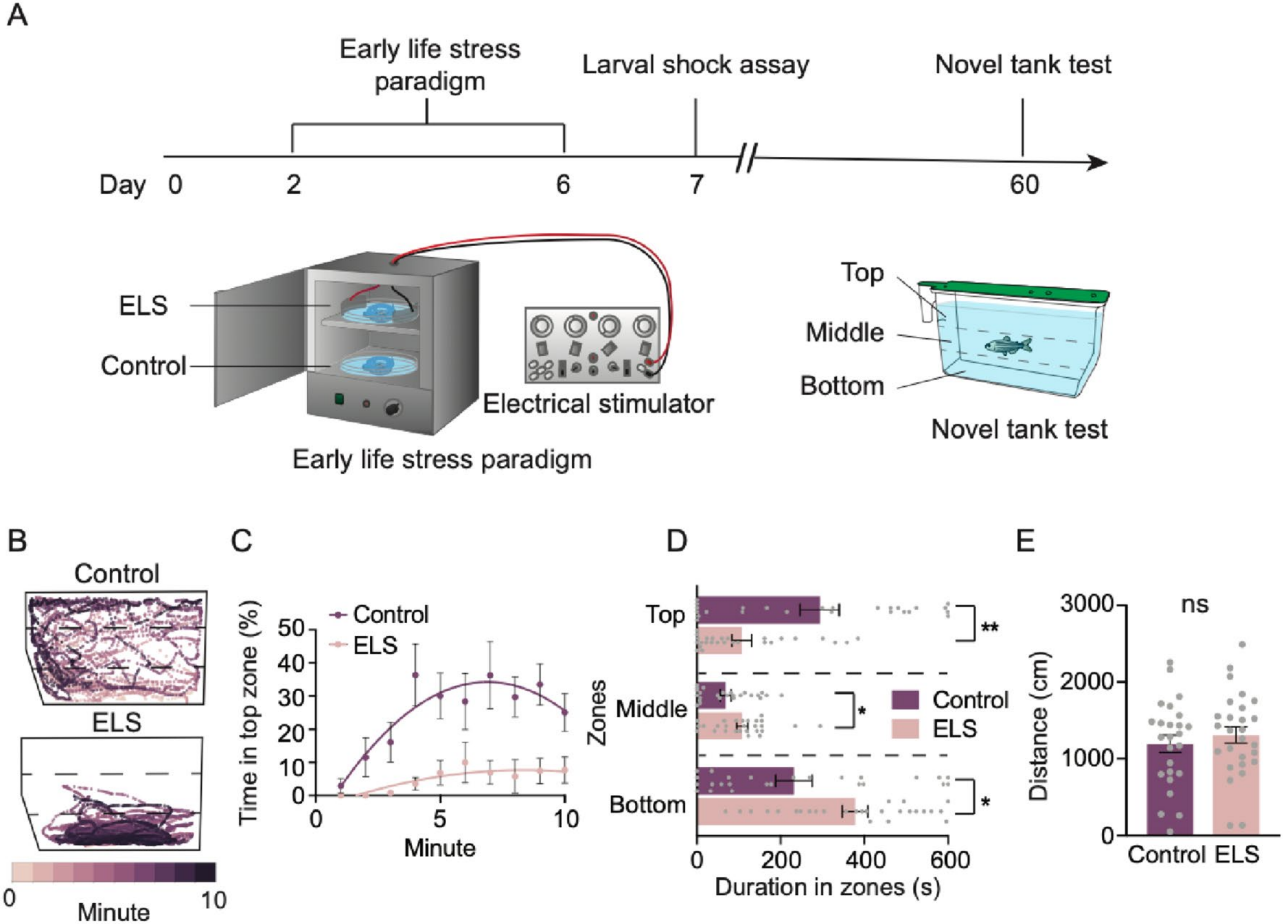
ELS results in exacerbated stress responses in adulthood. (**A**) Timeline of ELS and behavior experiments and illustrations of the ELS setup and novel tank test. (**B**) Representative swim trajectories of an individual control and ELS fish revealing that while control fish explored most of the tank, ELS fish swam more at the bottom of the tank. (**C**) Percentage of time spent in the top zone each minute throughout the 10-min recording. Over time, control animals gradually explored the top zone more frequently than ELS animals. (**D**) Compared to controls (n = 25), ELS adults (n = 27) spent less time exploring the top (Unpaired t test, p = 0.0003), and more time exploring the bottom (Unpaired t test, p = 0.0038) and middle (Unpaired t test, p = 0.018) zones. (**E**) Distances travelled were not different between controls (n = 25) and ELS (n = 27) animals (Unpaired t test, p = 0.46). Error bars show ± standard error of the mean. Asterisks denote statistical significance (*p = 0.05, **p = 0.005). ns denotes no significance.

To determine the long-term consequences of chronic stress in early development on zebrafish, wild-type (AB)^34^ larvae were subjected to random pulses of a mild electric current (25 V, 200 ms duration, 1 pulse per second) from 2 to 6 days post fertilization (dpf). This stimulus intensity was chosen as it was the minimum voltage required that caused more than 80% of 2 dpf larvae to react to shock. Moreover, we have previously shown this shock intensity causes a robust stress response in larval zebrafish^28^. Control siblings were handled similarly but were not subjected to electric shock. At 6 dpf, following cessation of the ELS protocol, all groups of larvae were transferred to a recirculating system in the main aquatics facility (Fig. 1A). The delivery of shock was random, and thus larvae were not able to predict stimulus onset (Fig. S1A). Video inspection revealed that larvae react to the electric shocks throughout the 5 day period (data not shown). We tested for behavioral differences to stress at larval (7 dpf) and juvenile (60 dpf) stages to determine whether this protocol has a lasting impact on stress response (Fig. 1A). Qualitative examination of swim bladder and locomotor behavior under a light microscope revealed that both control and ELS fish appeared healthy immediately following ELS at 7 dpf and after testing behavior at 60 dpf with no gross morphological abnormalities, and swimming in these animals was unaffected (Fig. S1B).

To determine if ELS leads to immediate changes in stress behavior, we quantified the differences in the behavioral response to stress between ELS and control animals at 7 dpf, one-day following cessation of shock (Fig. S1C). Exposure to mild electric shock causes freezing and reduced locomotion in zebrafish larvae, and the change in freezing and locomotion pre- and post-shock is a reliable measure of stress levels^28^. Control animals not subjected to shock in early life displayed significant freezing post-stimulation, revealing a stereotyped stress response in this group at 7 dpf. Unexpectedly, no significant differences in freezing pre- and post-stimulation at 7 dpf were observed in ELS groups (Fig. S1D). As freezing is an indicator of stress, these data suggest that ELS initially causes a reduction in stress responses in larval stages, potentially due to initial habituation. Freezing times in this assay were variable, however, and in some cases pre-freezing durations were high. Together, these data suggest that the behavioral effects of ELS may not be immediate, and may emerge only after developmental changes to the brain.

ELS in mammals has been shown to alter stress later in adult stages. To examine whether the effects of ELS are conserved across vertebrates, we measured stress responses in 60 dpf juveniles that were previously subjected to ELS. We tested fish in the novel tank test, which is widely used to quantify innate stress behaviors in juvenile and adult fish (Fig. 1A)^22,29^. When introduced into a novel tank, wild-type zebrafish initially prefer the bottom part of the tank, yet over time, they begin to explore the top part with higher frequency; the amount of time spent in the bottom portion of the tank has been validated as a behavioral indicator of stress^22,29,35^. At 60 dpf, control animals initially prefer the bottom zone of the novel tank, but by 4-min, they navigate all zones with high frequency (Fig. 1B,C). By contrast, adults previously subjected to ELS spent little time in top zone throughout the 10-min recording period (Fig. 1B,C). Quantifying total duration spent in all zones revealed that ELS animals spent significantly more time in the bottom zone of the tank over the 10-min recording period, and less time exploring the top zone compared to controls that were not exposed to ELS (Fig. 1D). Analysis of total distance moved and total duration immobile revealed no significant differences between control and ELS siblings (Fig. 1E & Fig. S1E), indicating that bottom dwelling durations were independent of locomotion or lethargy. These data suggest that ELS exposure during early development increases stress behavior in juvenile fish.

### Increased stress responses are accompanied by increases in basal cortisol levels and expression of stress-related pathway genes

**Figure 2:**
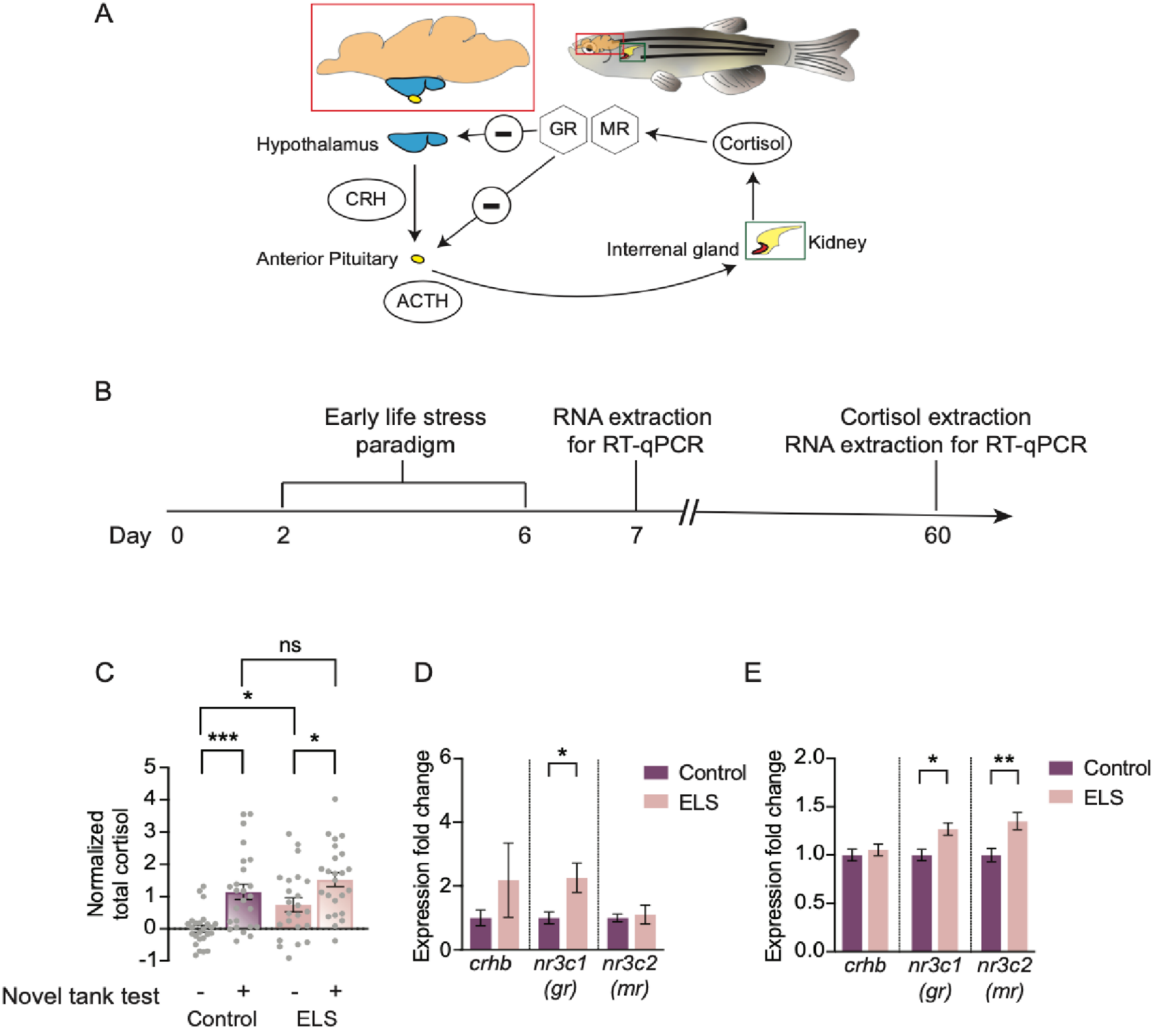
HPI axis is impacted in ELS. (**A**) The HPI axis, the main stress pathway, and its main genes and effectors. Brain of zebrafish is shown in the red bounding box. Within, the hypothalamus (in blue) and the anterior pituitary (in yellow) is shown. During stress, the hypothalamus signals to the anterior pituitary via CRH, and the anterior pituitary signals to the interrenal gland (shaded in red within the head kidney enclosed in the green bounding box) via ACTH. Cortisol is released from the interrenal gland and binds to GR and MR to negatively regulate its release. (**B**) Timeline of ELS and experiments performed. (**C**) Basal cortisol levels (−) were increased in ELS animals (n = 23) compared to controls (n = 25) (One-way ANOVA followed by Sidak’s multiple comparisons post-hoc test, p = 0.044). Elevated cortisol in response to stress (+), after the novel tank test, remain intact in control (n = 25, One-way ANOVA followed by Sidak’s multiple comparisons posthoc test, p = 0.00040) and ELS adults (n = 24, One-way ANOVA followed by Sidak’s multiple comparisons post-hoc test, p = 0.034). Cortisol levels after stress were no different between controls and ELS animals (One-way ANOVA followed by Sidak’s multiple comparisons post-hoc test, p = 0.54). (**D**) Quantitative real-time PCR of 7 dpf control (n = 9) and ELS (n = 8) larvae revealed increased gene expression levels of gr in ELS animals (Unpaired t test, gr: p = 0.018). No significant differences were found in expression levels of crhb and mr (Unpaired t test, crhb: p = 0.31, mr: p = 0.74). (**E**) At 60 dpf, gene expression levels of gr and mr were increased in brains of ELS animals (Unpaired t test, gr: p = 0.0064, mr: p = 0.0023), and no difference in crhb expression levels (Unpaired t test, p = 0.73) were observed, compared to controls. N = 9 per group. Error bars show ± standard error of the mean. Asterisks denote statistical significance (*p = 0.05, **p = 0.005, ***p = 0.0005). ns denotes no significance.

A hallmark of ELS in mammals is prolonged or increased circulating cortisol in the plasma, which correlates with exacerbated stress responses^9,36–39^. We next asked if ELS resulted in changes in the zebrafish neuroendocrine HPI axis. In fish, environmental stressors activate a highly conserved cascade of events from the brain to peripheral tissue to produce and release cortisol (Fig. 2A). To determine whether ELS induced alterations in cortisol and other molecules in the HPI axis, we subjected larvae to random shock from 2 to 6 dpf, raised animals to 60 dpf, and measured both baseline and stress-induced whole-body cortisol levels after being challenged in the novel tank test (Fig. 2B). Compared to controls, basal levels of cortisol were significantly increased in ELS animals (Fig. 2C). By contrast, no differences were observed between ELS and control animals after the novel tank test (Fig. 2C). In both ELS and control siblings, cortisol levels increased significantly following testing in the novel tank test (Fig. 2C), suggesting that both groups have an intact physiological response to stress. These data demonstrate that ELS leads to chronically elevated cortisol production.

To further examine how ELS impacts the physiological response to stress, we measured mRNA transcript abundance of several genes in the HPI pathway. Environmental stressors activate *corticotropic releasing hormone* (*crh*) neurons in the hypothalamus^40^. CRH then signals indirectly to the interrenal gland to synthesize and release cortisol, which activates mineralocorticoid (mr) and glucocorticoid receptors (gr) in the brain (Fig. 2A)^41^. To test whether ELS altered the abundance of transcripts in the HPI pathway, larvae were subjected to chronic stress from 2 to 6 dpf, and then at either 7 or 60 dpf, brains were dissected, and transcript abundance was measured for *crhb*, *nr3c1* (glucocorticoid receptor; *gr)*, and *nr3c2* (mineralocorticoid receptor; *mr*) using quantitative real-time polymerase chain reaction (qRT-PCR; Fig. 2A,B). Quantitative gene expression analysis revealed elevated levels of *gr* in ELS-treated animals compared to control siblings at 7 dpf (Fig. 2D), and its expression levels remained significantly higher than controls at 60 dpf (Fig. 2E). Furthermore, at 60 dpf, expression levels of *mr* were also elevated in ELS animals (Fig. 2E). By contrast, no significant differences were found for the relative expression of *crhb* (Fig. 2D,E) suggesting that its broad expression in stress-related regions such as the ventral hypothalamus and preoptic area of the hypothalamus, remains unchanged. Therefore, ELS results in lasting changes in the HPI axis at the transcriptional and physiological levels.

### Enhanced stress following pharmacological activation of the cortisol pathway

**Figure 3:**
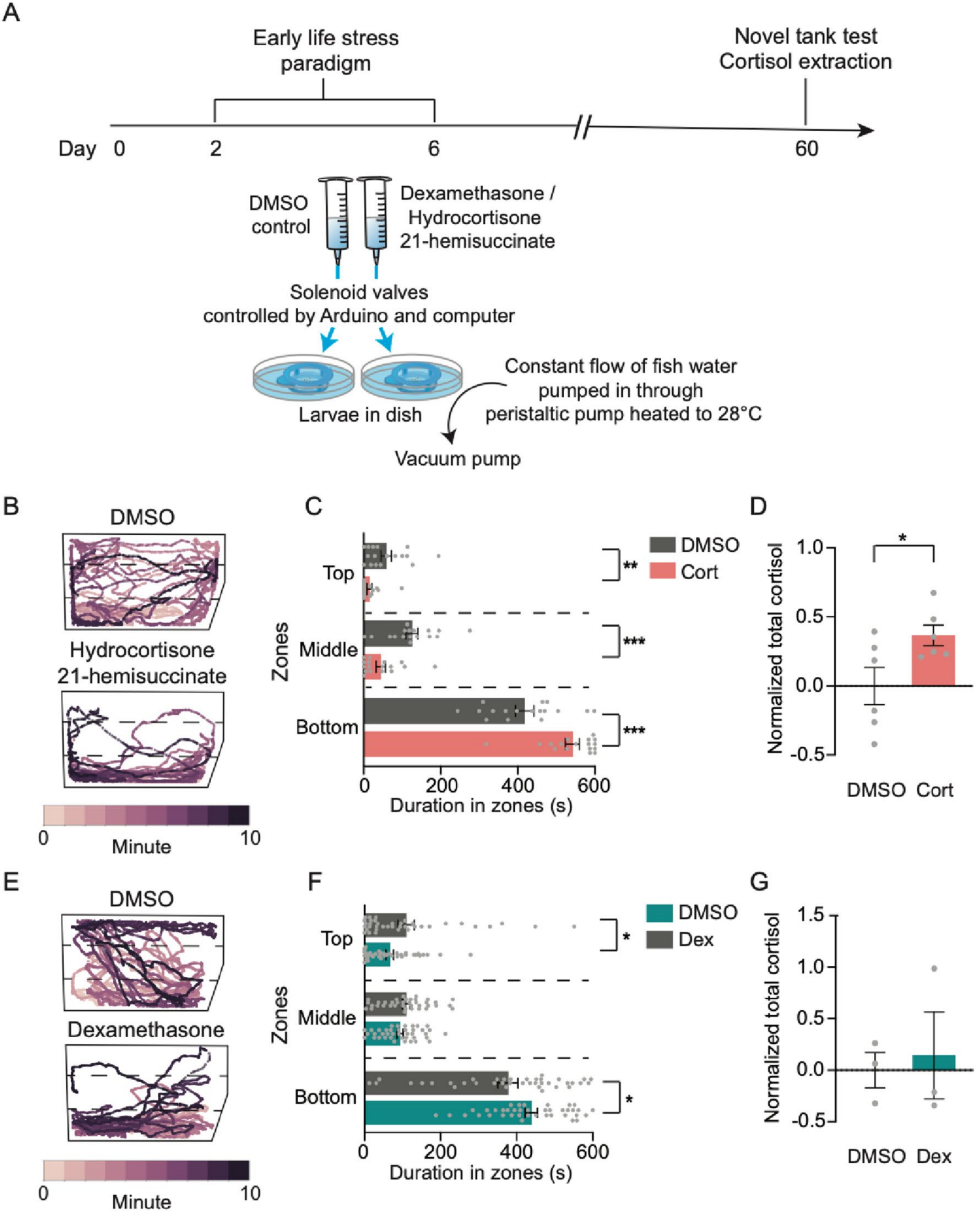
Treatment with corticosteroid receptor agonists in early life induces adulthood anxiety. (**A**) Timeline of drug-treated ELS and experiments conducted. A schematic diagram of the setup is presented below the timeline. (**B**) Representative swim paths of 60 dpf controls (DMSO) and hydrocortisone 21-hemisuccinate- (Cort) treated animals. Drug-treated individuals tend to spend more time at the bottom of the tank. (**C**) Total duration spent in the bottom zone of the novel tank was significantly increased in Cort animals (Unpaired t test, p = 0.0001), and decreased in the top (Unpaired t test, p = 0.0026) and middle (Unpaired t test, p = 0.0002) zones, compared to control DMSO. N = 17 per group. (**D**) Basal cortisol levels were significantly higher in Cort animals than control DMSO siblings (n = 6 per group, Unpaired t test, p = 0.038). (**E**) Representative swim paths of 60 dpf controls (DMSO) and dexamethasone- (Dex) treated animals. Drugtreated individuals tend to spend more time at the bottom of the tank. (**F**) Quantification of durations spent in top, middle, and bottom zones of the novel tank test revealed that Dex animals spent more time in the bottom zones (Unpaired t test, p = 0.025), and less time in the top (p = 0.035) than control DMSO animals yet no difference was observed in time spent in the middle (Unpaired t test, p = 0.11). DMSO: n = 38; Dex: n = 40. (**G**) Measurements of basal cortisol levels were no different between Dex and DMSO animals (n = 3 per group, Unpaired t test, p = 0.77). Error bars show ± standard error of the mean. Asterisks denote statistical significance (*p = 0.05, **p = 0.005, ***p = 0.0005). ns denotes no significance.

In mammals, increased glucocorticoid signaling is strongly correlated with enhanced stress response later in life, yet whether increased glucocorticoids are sufficient to cause enhanced stress in adult stages is unclear. The small size and *ex utero* development of zebrafish has made this system a powerful model for screening of pharmacological compounds^42–44^. To test whether elevated glucocorticoid signaling alone is sufficient to induce long-term changes in stress responses, we pharmacologically activated glucocorticoid signaling between 2 and 6 dpf, and measured whether this was sufficient to cause increased anxiety later in juvenile fish. Glucocorticoids were provided in a continuous flow system and boluses of drug were delivered to larvae randomly using the same protocol we used for delivering electric shock in early life (Fig. 3A).

Juvenile animals dosed with the synthetic glucocorticoid that binds to both MR and GR hydrocortisone 21-hemisuccinate (Cort) in early life spent increased time in the bottom and less time in the top of the novel tank compared to undosed sibling controls (Fig. 3B,C). Likewise, we observed an increase in basal levels of cortisol in Cort-treated animals compared to controls (Fig. 3D). Locomotor activity was reduced in Cort-treated animals (Fig. S3A), but this is unlikely to be attributed to a general loss of coordination because the total time spent immobile did not differ from control siblings (Fig. S3B). Taken together, these findings reveal that pharmacological activation of the MR/GR pathways phenocopy shock-induced ELS.

We next asked if overactivation of GR alone was sufficient to phenocopy ELS through the application of dexamethasone (Dex), a selective glucocorticoid agonist^45,46^. Dex-treated animals spent more time in the bottom of the novel tank and less time in the top and middle zones compared to control animals (Fig. 3E,F). Interestingly, basal cortisol levels were not significantly different between Dex-treated and control animals (Fig. 3G), suggesting cortisol levels may be separable from the stress response.

Distance travelled and duration of immobility did not differ between control DMSO- and Dex- treated animals (Fig. S3C,D). Taken together, these data suggest that ELS alters brain development through dysregulation of GR signaling.

### A critical window for increased anxiety following ELS associates with HPI development

**Figure 4:**
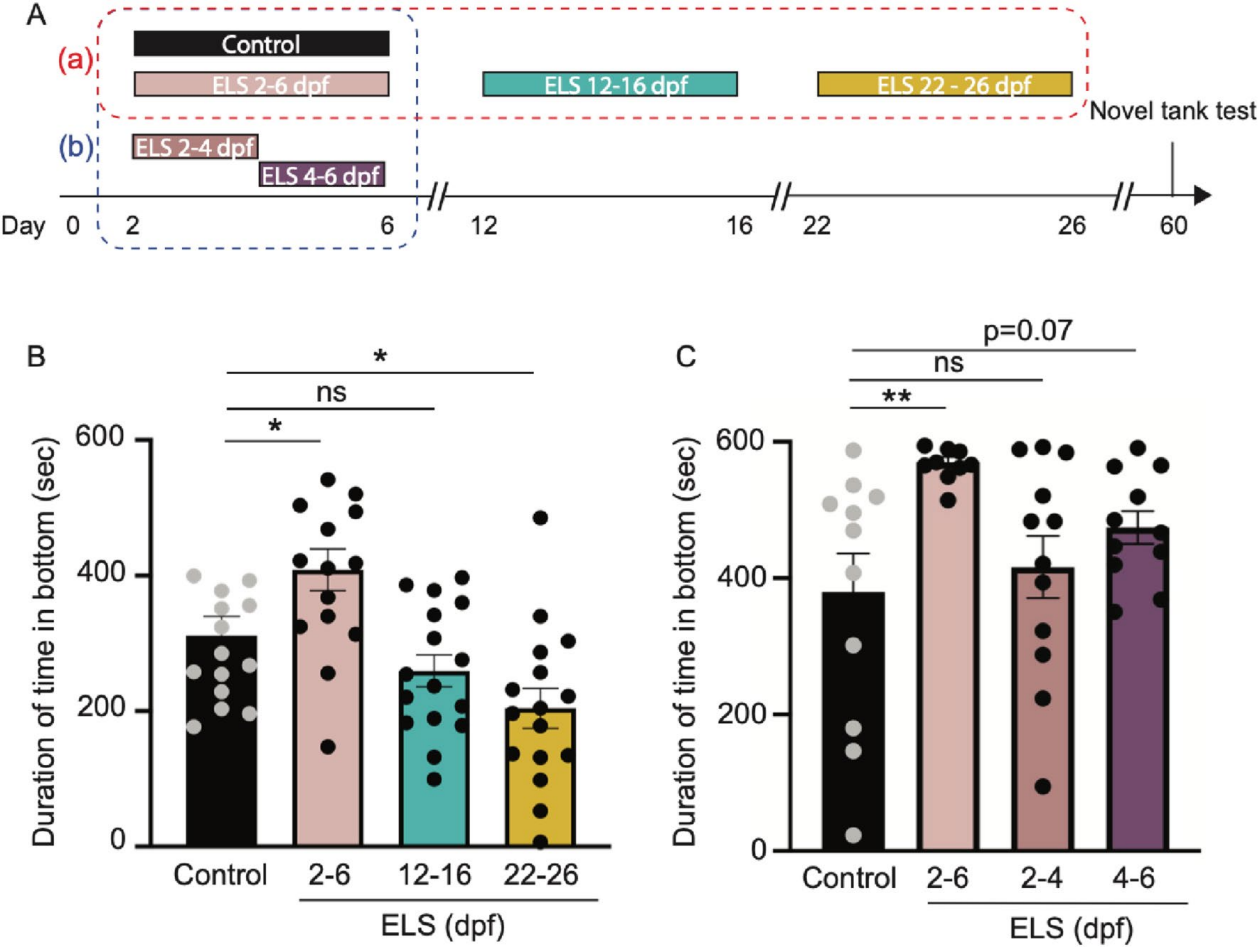
Chronic stress in a time window between 4 and 6 dpf is critical to impact behavior later in life. (**A**) Timeline of ELS paradigm and novel tank test. Different groups of larvae were subjected to ELS at different ages. In group (a) (red box), larvae were placed in ELS paradigm at 2–6, 12–16, or 22–26 dpf, alongside same aged and unshocked controls. In group (b) (blue box), larvae were placed in the paradigm at 2–6, 2–4, or 4–6 dpf, while control siblings remained alongside in the incubator throughout the 5 days. (**B**) Quantification of duration spent in the bottom zone of the novel tank test at 60 dpf suggest that only chronic stress during the 2–6 dpf period caused increased bottomdwelling behavior, and not at later times. Multiple unpaired t-tests were performed between control and ELS siblings at three time windows. Control vs. ELS 2–6 dpf : p = 0.014; Control vs. ELS 12–16 dpf: p = 0.081; Control vs. ELS 22–26 dpf : p = 0.0066. (**C**) Quantification of durations spent in the bottom zone of the novel tank suggest that stress between 4 and 6 dpf may be sufficient to cause increased bottom-dwelling behavior later in life. Statistical analysis done using multiple unpaired t-tests were performed between control and ELS siblings at three time windows. Control vs. ELS 2–6 dpf: p = 0.0026; Control vs. ELS 2–4 dpf: p = 0.31; Control vs. ELS 4–6 dpf: p = 0.07. Controls: n = 11; ELS 2–6 dpf: n = 10; ELS 2–4 dpf: n = 12; ELS 4–6 dpf: n = 11. Error bars show ± standard error of the mean. Asterisks denote statistical significance (***p = 0.0005, **p = 0.005, *p = 0.05).

Chronic stress in mammals and birds during specified time windows has lasting effects on brain development and function, yet whether this extends to other vertebrates is poorly understood^7,9,47^. To identify whether ELS impacts later stress response through a developmentally sensitive time window, wild-type animals were subjected to the same ELS paradigm described above at varying time periods throughout development (Fig. 4A). Fish were subjected to ELS for a 5-day period in the first, second or third week of life (i.e., from 2 to 6 dpf, 12–16 dpf, or 22–26 dpf) and the effects on stress behavior were measured at 60 dpf (Fig. 4A (a), red box). Whereas chronic stress from 2 to 6 dpf resulted in enhanced bottom dwelling at 60 dpf, increased bottom-dwelling behavior was not observed when fish were subjected to the ELS paradigm at either 12–16 dpf or 22–26 dpf (Fig. 4B; Fig. S4A), suggesting wildtype zebrafish are sensitive to ELS in a time window between 2 and 6 days after fertilization. Interestingly, fish subjected to chronic shocks from 12 to 16 and 22–26 dpf had reduced bottom dwelling, suggesting that shock at these late stages either reduced stress levels^48^, or fish habituated to stress from the shocks.

To define the time window between 2 and 6 dpf when ELS induces long-term effects on stress response, we subjected larvae to the ELS paradigm described above from 2 to 6 dpf, or from a more restricted 2–4 or 4–6 dpf time window. Fish were then raised to 60 dpf and tested in the bottom dwelling assay (Fig. 4A (b), blue box). Consistent with previous data, larvae subjected to chronic stress from 2 to 6 dpf showed significant increases in the total time spent in the bottom of the tank when tested at 60 dpf (Fig. 4C). By contrast, no significant effects were observed in zebrafish subjected to shock from 2 to 4 dpf (Fig. 4C). Instead, larvae subjected to chronic shock from 4 to 6 dpf showed a trend towards increased bottom dwelling behavior when examined at 60 days (Fig. 4A,C; Fig. S4B, p = 0.07). The effect of chronic stress from 4 to 6 days was less pronounced than when fish were shocked from 2 to 6; thus, while the main effect is likely in the 4–6 dpf time-period, these data cannot rule out other potentially contributing factors. Together, these data suggest that zebrafish larvae are sensitive to ELS in a critical window after the neuroendocrine stress axis is functional and zebrafish are synthesizing their own cortisol, but before brain development is complete^30,49,50^.

## Discussion

Our findings demonstrate that zebrafish, like mammals, have augmented stress responses when subjected to ELS early in development. We also show that the negative effects of ELS act, in part, through chronic activation of GR, whose expression changes over the course of development^30^. Notably, enhanced stress following ELS was not observed in the day following ELS, but rather enhanced stress emerged later, further supporting the notion that ELS is impacting development of the brain. The critical window for chronic stress is after the time where the neuroendocrine stress axis is formed^49^, suggesting that ELS is not acting directly on the development of the HPI axis, but rather on hormonal signaling to the brain.

In humans, childhood trauma, such as abuse or parental neglect, has potent impacts on adult behavior, stress responsivity, susceptibility to develop stress-related disorders, and substance abuse issues^51–53^. Moreover, genome-wide association studies have identified components of glucocorticoid-mediated gene regulatory networks suggesting ELS may alter the expression of this pathway during brain development. Genetic manipulation of the glucocorticoid receptor in early life, but not later life, also leads to enhanced stress-related pathologies in later life, supporting the notion that glucocorticoid receptor signaling in early development is a critical mediator of ELS^54,55^. However, an analysis of how abnormal glucocorticoid signaling alters the development of the brain has been challenging in mammalian systems. Our findings reveal the molecular basis of ELS is highly conserved and provide a powerful genetic model for investigating the mechanistic basis of ELS on neurodevelopment.

### A new model of ELS

Here, we demonstrate that zebrafish display significantly elevated stress responses when subjected to chronic stressors in larval stages, similar to what is found in mammals and other studies examining fish^21,48,56–60^. This suggests zebrafish may be a unique and powerful complement to mammalian systems, especially for studying how ELS causes changes to neurodevelopment. Zebrafish have several attributes that make them an attractive complement to mammalian models in the study of ELS. Originally established as a model in development, zebrafish fertilization and development are external and fish embryos are transparent^61,62^. The transparency not only permits examination of all stages of development, but also highlights the power of the system in circuit neuroscience^63,64^. This work sets the stage for using powerful tools, such as whole-brain functional imaging and genetic manipulation of precise neuronal subsets, to study how brain anatomy and function change over the course of development in response to ELS^65–68^.

Several paradigms have been developed in zebrafish for challenging fish at larval stages and examining the consequences in later life^69,70^. One common approach has been to treat fish chronically with cortisol^21,58^ or other glucocorticoid agonists^71^. Early exposure to these agonists result in changes in locomotion^58^, enhanced expression of immunological markers, and increased bottom dwelling in the novel tank assay^71,72^, though the effect on bottom dwelling has been challenged^33^. Several other assays with more ethological relevance have also been used, including a restraint paradigm, where fish are affixed to the bottom portion of a 6-well plate from 3 to 9 dpf, a forced swim assay using a stir bar to induce locomotion from 4 to 8 dpf, and a chronic unpredictable early life stress (CUELS) assay using several manipulates to light, temperature and density^21,56,57,73,74^. In these cases, early life disruptions result in enhanced measures of stress, though behavioral assays used to study stress differ among studies. Our data are consistent with these findings, and introduce a new paradigm in the field of early life stress.

A significant advantage to studying early life stress in zebrafish is the accessibility to the system at different developmental time points, and the ability to easily restrict early life disruptions to different time periods and determine the critical period. Our data reveal that fish are most sensitive to ELS from 4 to 6 days post fertilization. In mice, the stress hyporesponsive period is from post-natal days 1–10 (P1-10) whereas in rats it is P4-14^75–78^. The SHRP in fish is not clear, yet one study suggests that fish are able to produce cortisol at 3 dpf, but only to “life threatening” (i.e., potentially harmful) stimuli, and that fish emerge from their SHRP by 5 dpf^30^, while other studies failed to see any changes at 5 dpf^70^. Thus, while fish do appear to have a SHRP, the exact timing of the period is unclear. Our data show that fish are most sensitive to ELS from 4 to 6 days, which coincides with fish emerging from their SHRP. How this impacts the sensitivity to ELS, or whether chronic unpredictable shocks represent a potentially harmful stimulus or not is unclear, yet future data examining glucocorticoid responsiveness with tools unique to zebrafish^79^ may help to clarify this phenomena. Interestingly, fish subjected to chronic shocks from 12 to 16 dpf and 22–26 dpf had reduced bottom dwelling relative to controls, suggesting that these later stage disruptions may lead to less anxiety-like behavior. This is consistent with other reports in zebrafish^48,60^. It could be therefore that ELS in this context results in long-term changes and enhanced anxiety-like behavior, whereas late-stage stressors may lead to stress resilience.

### The role of GR in enhanced stress following ELS

Our data suggest that both ELS and cortisol exposure lead to enhanced levels of baseline cortisol. Interestingly, mimicking ELS with Dex, a potent synthetic GR agonist resulted in enhanced levels of stress but no differences in cortisol. It is unclear why baseline cortisol levels were increased when ELS was mimicked with Cort and not Dex, but one possible explanation is that Dex is a stronger agonist of the GR receptor relative to Cort. Glucocorticoid signaling inhibits crh-neurons in the hypothalamus, thereby causing a negative feedback loop. Moreover, while zebrafish produce cortisol, Dex is a synthetic activator of GR singling, and previous studies in zebrafish have shown that treatment of Dex result in lower levels of cortisol^33^. Therefore, it could be that the strength of Dex, combined with a lack of endogenous activity and loss of possible compensatory mechanisms, could overpower the neuroendocrine stress axis, and cause a strong inhibition of its activity.

Several hypotheses exist to explain how enhanced cortisol activation can impair development. A prevailing model suggests that the effects of ELS emerge through an imbalance in the relative proportion of MR:GR^80–82^. Our data do not support this hypothesis in zebrafish. While the expression of both GR and MR were higher in ELS-subjected animals, the relative balance, or ratio, was not significantly different (Fig. S2). One explanation for this result is that there may be functional differences in the response of these receptors, and while the stoichiometry of the expression, or the ratio of their mRNA levels, may not be different^82^, their function may be. The glucocorticoid receptor, for example, has several isoforms. The GRα isoform is highly active while the GRβ is inert and can act as an inhibitor of GR signaling^83–85^. Alternatively, overactivation of GR alone may impair brain development. In mammals, chronic activation of GR results in neuronal death, dendritic spine retraction, and reduced spike frequencies^86–92^. Moreover, overexpression of GR throughout the life of the rodents, or transient activation of GR only in early development is sufficient to lead to enhanced anxiety, whereas transient activation in adult stages has no effect^55^. Our data thus support a conserved role of dysregulated GR signaling in enhanced risk, and point to an ancient origin for GR signaling in the brain and its contribution to stress disorders.

### Neurodevelopmental implications of ELS in zebrafish

A central strength of the zebrafish in the study of ELS is the ability to interrogate the effects of stressors at specific developmental time points, and associate those time points that impact stress to precise developmental processes. This central strength reveals several findings about ELS. First, our data reveal that long lasting effects of chronic stress emerge only when stressors are given in early time points. Thus, enhanced stress following ELS is likely not a passive response to elevated stress but rather ELS likely impedes normal brain development. Moreover, that larvae are particularly sensitive to stressors from 4 to 6 dpf, and less so at 2–4 dpf, points to specific developmental processes that may be impacted.

The HPI axis begins forming early in development, and expression levels of both GR and MR show significant fluctuations until approximately 2 dpf; by 49 h post fertilization, expression levels of GR in normal reared animals are stable. Zebrafish do not begin to produce cortisol in response to exogenous stressors until 4 dpf^30^. Because the HPI axis is functional by 4 dpf, our data suggest that ELS is not impacting the development of the neuroendocrine stress axis. Moreover, as larvae are particularly sensitive to ELS from 4 to 6 dpf, a time point when animals are beginning to produce cortisol, it is likely that ELS is leading to overactivation of glucocorticoid signaling, which in turn impacts brain development. While the critical period of ELS in humans is unclear, human fetuses begin to produce their own cortisol by eight weeks of gestation^93^, and previous studies have shown that prenatal infants are also susceptible to chronic stress experienced by pregnant mothers^94^. These data could therefore suggest that vertebrate animals are susceptible to the impacts of ELS at a time after they produce their own cortisol and no longer rely on cortisol from maternal load.

Significant changes in brain development also occur during the 4–6 dpf window. In the zebrafish forebrain, a large neuroanatomical region with loci analogous to the mammalian limbic system, newborn neurons begin to form by 2 dpf^95^. By 4 dpf, development of the zebrafish forebrain is complete and neuronal properties such as spontaneous activity and neurotransmitter identity are beginning to develop^96^. Significant change in neuronal activity also occur in these early time points. Spontaneous activity in the zebrafish brain is observed beginning from 2 dpf through adulthood, yet significant changes emerge over developmental time. In the tectum, spontaneous activity is random and disorganized at 2 dpf, yet by 8 dpf, the circuit is functional mature, and clusters of functionally relevant neurons fire in unison^97,98^. These data support the notion that development and function of the neuroendocrine stress axis are impacted by ELS. Future studies utilizing transgenic and neuroimaging techniques on this model of ELS will reveal how specific changes in brain development and maturation of neuronal activity following ELS lead to enhanced stress responses in later life.

## Conclusions

Our data introduce a new model in the field of early life stress, and uncover several principles about how ELS may impact the developing brain. The zebrafish is a strong model in developmental biology and neuroscience, and thus the unique combined strengths of this model provide unprecedented insight into how the brain responds to stress in these early time periods. Zebrafish exhibit stereotyped and well-studied stress responses and there are a number of assays available to examine stress in fish. Moreover, the high conservation at the neuronal and physiological levels between fish and humans suggest that findings in zebrafish will translate well to the mammalian system, and should complement mammalian work. Furthermore, the large collection of mutant and transgenic lines available in zebrafish, optical approaches for monitoring brain development in vivo, computational tools such as the recently developed brain atlases, and whole-brain functional imaging all coalesce and provide a single model that uniquely bridges development and neuroscience.

## Material and methods

### Ethics statement

All experiments in this study conformed to and were approved by the Institutional Animal Care and Usage Committee at Florida Atlantic University, protocol number A17–22, and were in accordance with ARRIVE guidelines (http://arriveguidelines.org)^99^. All methods were performed in accordance with the relevant guidelines and regulations set forth by the National Institutes of Health and the guidelines for animal research at Florida Atlantic University.

### Animal care

Wild-type AB^34^ zebrafish were used in all experiments in this study. Adults were kept in 1.8–10 L tanks on a recirculating aquatics system (Aquaneering). The water temperature was maintained at 28 ± 1 °C, and the light cycle was set to a 14:10 light:dark cycle. In order to breed larvae, 4–6 adult breeder fish (half males and half females) were placed in a mating tank (1L, ZHCT100, Aquaneering), together with some plastic plants, and allowed to spawn naturally overnight. The morning after spawning, eggs were collected, placed in 100 × 15 mm Petri dishes (Fisherbrand, Fisher Scientific Inc.), and housed in an incubator (Heratherm IMC18, Thermo Scientific) set at 28 ± 1 °C. Fish subjected to ELS, as well as their control siblings, were housed on the same re-circulating system, in dedicated tanks containing 500 mL of 5 ppt salt water, co-cultured with L-type rotifers from 6 to 15 dpf and fed with GEMMA Micro 75 (Skretting Zebrafish) daily. At 15 dpf, the valve was opened to allow system water to flow at a slow rate into the tanks, rotifers were washed out, and fish transitioned to be fed *Artemia* (brine shrimp) and GEMMA Micro 150 (Skretting Zebrafish). Fish older than 30 dpf were fed GEMMA Micro 300 (Skretting Zebrafish) daily. Experiments were carried out between 11 a.m. and 6 p.m., and according to a protocol (A17–22) approved by the Institutional Animal Care and Use Committee of Florida Atlantic University.

### ELS paradigm

Early life stress was induced in zebrafish larvae using a custom written computer program (MATLAB R2019a, MathWorks). This script controlled a square pulse stimulator (SD9 Grass Stimulator, Grass Technologies Inc) interfaced through a microcontroller board (Arduino Uno R3, Arduino) to randomly deliver electric current to fish from 2 to 6 dpf. Groups of thirty to fifty 2 dpf larvae were placed in 40 µm cell strainers (Corning Inc.) placed in 100 × 15 mm Petri dishes (Fisherbrand, Fisher Scientific Inc.) filled with fresh system water. A dish containing control larvae, which were not subjected to ELS, as well as a dish containing larvae that were subjected to ELS were placed in an incubator at 28 °C on a 14:10 light:dark cycle. A pair of electrodes connected to the square pulse stimulator was placed on opposite ends of the dish containing larvae to be subjected to ELS. The stimulator was controlled by a microcontroller board (Arduino Uno R3, Arduino), which was connected to a computer (Inspiron 15 3000 series, Dell). The custom written program was designed to evaluate a random number between 0 and 1 every ten minutes. In cases where the random number was greater than or equal to 0.5, 5-electric pulses were delivered (25 V, 1 Hz, 200 ms pulse duration), effectively delivering shocks 50% of the time; by contrast, if the random number was less than 0.5, no stimulation was provided. The net effect of this program was random stimulation so that fish were not able to predict. Fish were subjected to this protocol from 2 to 6 dpf. At the end of the 6 dpf, fish were removed from the incubator, and placed on the recirculating system where they were raised to 60 dpf. For critical time windows experiments, fish at 2 dpf, 12 dpf, and 22 dpf were subjected to the ELS paradigm as described above for five days, except that for 12 and 22 dpf fish, twenty juveniles were placed in glass Pyrex bowls (470 mL, Pyrex) and co-cultured with L-type rotifers and fed with GEMMA Micro 75 (Skretting Zebrafish) from 12 to 16 dpf, and GEMMA Micro 150 (Skretting Zebrafish) from 22 to 26 dpf.

### Pharmacological induction of ELS

ELS was induced pharmacologically using a modification of the ELS paradigm described above. Thirty 2 dpf larvae were placed in 40 µm cell strainers (Corning Inc.) housed in in petri dishes (50 × 15 mm, Fisherbrand, Fisher Scientific Inc.). Petri dishes were placed in an enclosed space, and were connected to a flowthrough, temperature-controlled water delivery system, consisting of a peristaltic pump (EW-78001-60, Cole-Parmer), which delivered fresh system water at a rate of 30 µL/s, a pair of heating/cooling cubes (ALA Scientific Instruments) that maintained water temperature at 28 ± 1 °C, and a vacuum pump (ALA Scientific Instruments), which maintained water volume of 12 ± 1 mL in the dishes. Using the same computer program and microcontroller board previously described, two solenoid valves connected to dosing syringes were programmed to deliver either 1.5 mL of 0.1 mM dexamethasone (PHR1526, Sigma-Aldrich), 25 µM hydrocortisone 21-hemisuccinate (H4881, Sigma-Aldrich), or control 1% dimethyl sulphoxide (D8418, Sigma-Aldrich) into each petri dish. The treatments were delivered at 50% chance at every 10 min interval. Similarly, at the end of 6 dpf, the larvae were placed back into the facility and raised to 60 dpf.

### Larval shock assay

At 7 dpf, a day after the ELS paradigm, the shock assay was performed as previously described^28^ to analyze stress behavior. Briefly, a single larva was placed in a 40 µm cell strainer (Corning Inc.) that was on a 3 cm raised platform in a 6 × 6 × 6 cm (L × W × D) container. On opposite sides, two electrodes were connected to a stimulator (SD9 Grass Stimulator, Grass Technologies Inc.). The bottom of the container was illuminated by infrared Light Emitting Diodes (LED; 880 nm) and a white acrylic board that acted as a diffuser. Overhead, a high framerate cMOS camera (Grasshopper3, PointGrey, FLIR Integrated Imaging Systems, Inc.) captured larval behavior at 120 fps for 2 min and 5 sec—a minute of normal swimming behavior, followed by five seconds of electric shock at 1 pulse-per-second at 25 V, and then a minute post-shock. Records of each trial were captured using FlyCap2 Software (version 2.11, PointGrey, FLIR Integrated Imaging Systems, Inc.; https://www.flir.com/support-center/iis/machine-vision/downloads/spinnaker-sdk-flycapture-and-firmware-download/). Tracking of individuals were done offline in EthoVision XT (v13, Noldus), and each frame was inspected manually for inaccurate tracking, which were fixed in Ethovision. Raw XY coordinates were exported and a custom-written script in MATLAB (R2019a, MathWorks) was used to analyze ‘freezing’ behavior. Freezing was determined as immobility for more than 1.99 secs, as previously described^28^. Larvae used for this behavioral assay were euthanized and not used in adult tests and analyses.

### Novel tank test

60 dpf (± 3 days) control and experimental adults were transported in their home tanks from the fish facility to the behavior room and acclimated for an hour before the behavior test. An adult was first subjected to a stressor of being removed out of water for 3 min with a net. Then, the individual was allowed to recover briefly for 10 min in a 250 mL beaker filled with 200 mL fresh system water. Following the rest period, the individual was gently poured into a 1.8L novel tank (ZT180, Aquaneering) positioned with custom designed infrared Light Emitting Diodes lights (LED; 880 nm) in the background, and recorded for 10-min in front of a high-frame-rate cMOS camera (Grasshopper3, PointGrey, FLIR Integrated Imaging Systems, Inc.). Records of each trial were captured using FlyCap2 Software (version 2.11, PointGrey, FLIR Integrated Imaging Systems, Inc.; https://www.flir.com/support-center/iis/machine-vision/downloads/spinnaker-sdk-flycapture-and-firmware-download/). Tracking of individuals and analyses of durations spent in zones were done offline in EthoVision XT (v13, Noldus), and each frame was inspected manually for inaccurate tracking. The height of the water level was divided equally into three zones to analyze the durations spent in each zone. Durations spent in each zone, distance travelled, duration of immobility, and XY coordinates were obtained from the software. From XY coordinates obtained, swim trajectories were plotted using matplotlib in Python (v3.7).

### Cortisol measurements

To obtain basal cortisol, 60 dpf adults were flash frozen in individual 1.5 mL Eppendorf tubes. Post-stress cortisol was obtained from adults immediately after the novel tank test. Frozen samples were kept at − 20 °C for up to a month before cortisol extractions. Whole adult fish were weighed, then homogenized, and cortisol was extracted following Cachat et al.^22^ with minor modifications. Briefly, each individual was homogenized in 500 µL phosphate buffered saline (PBS). Then, another 500 µL PBS was added before decanting the sample into a glass scintillation vial. To extract cortisol, 2 mL diethyl ether (E134, Fisher Scientific) was added to the homogenate. Next, the samples were vortexed and then centrifuged. Subsequently, the organic diethyl ether layer containing cortisol was extracted into a new glass vial. The extraction steps were performed three times. After evaporation of the organic layer, 1 mL PBS was added, and the sample was kept at 4 °C overnight. The ELISA assay (#1-3002, Salivary Cortisol ELISA Kit, Salimetrics, LLC) measuring the amounts of cortisol was performed the following day. A standard dilution curve was made with standards provided. Total cortisol was normalized to body weight. Then, to account for daily variations in extractions and variations in kits, cortisol levels were normalized again to the mean control basal cortisol levels for each day using the following formula:

$$\text{Total Cortisol = }\frac{\left(\text{Cort - }\widehat{{\text{Cort}}_{\text{cntrl}}}\right)}{\widehat{{Cort}_{cntrl}}}$$

where $Cort$ represents total baseline cortisol levels, and $\widehat{{Cort}_{cntrl}}$ indicates the average cortisol levels measured for each day.

### Quantitative gene expression analysis of HPI axis genes

For gene expression analysis at 60 dpf, brains were dissected and immediately snap frozen in liquid nitrogen. Three brains were pooled together for each biological replicate, and were kept in − 80 °C for RNA extraction the following day. RNA was extracted using TRIzol (Thermo Fisher Scientific) and the RNeasy mini kit (QIAGEN). Genomic DNA was removed by DNase treatment (RNase-free DNase set, QIAGEN). 1000 μg of RNA was reverse transcribed into cDNA (iScript cDNA Synthesis Kit, Bio-Rad), and the subsequent cDNA was diluted to a concentration of 50 ng/μL to use for quantitative real-time PCR (CFX96 Touch Real-Time PCR Detection System, Bio-Rad). For gene expression analysis at 7 dpf, groups of twenty larvae were used for each biological replicate. Target gene expression levels were normalized to actin beta-1 and tubulin alpha-1c. Refer to Table S1 for primer sequences.

### Quantification and statistical analysis

Statistical analyses were performed using Prism 8 (v8.0.2, GraphPad Software; http://www.graphpad.com). Parametric tests were used unless the data failed the Shapiro–Wilk normality test, then non-parametric tests were used. For pairwise comparisons between control and ELS groups, one-tailed unpaired t tests were used. The non-parametric equivalent, Mann Whitney test was used when the data failed the normality test. Where comparisons were made between multiple groups, one-way ANOVA was performed, and when statistical significance between groups was obtained, the Sidak’s multiple comparisons post-hoc test was performed. The non-parametric equivalent of the one-way ANOVA used was the Kruskal–Wallis test, followed by the Dunn’s multiple comparisons post-hoc test.

## Supplementary Information

Supplementary Figure S1.

Supplementary Figure S2.

Supplementary Figure S3.

Supplementary Figure S4.

Supplementary Table S1.

## Data Availability

The datasets generated and/or analyzed during the current study are available from the corresponding author upon reasonable request. Cartoon diagrams in Fig. 1a and Fig. S1b were drawn in our lab.
