# Supplementary material for: Long lasting anxiety following early life stress is dependent on glucocorticoid signaling in zebrafish
Source: Sci Rep. 2022 Jul 27;12:12826. doi: 10.1038/s41598-022-16257-5 (PMC9329305; doi:10.1038/s41598-022-16257-5)
Supplement: Supplementary file 1 — Supplementary Figure S1. [file 41598_2022_16257_MOESM1_ESM.pdf]

Supplemental Figures

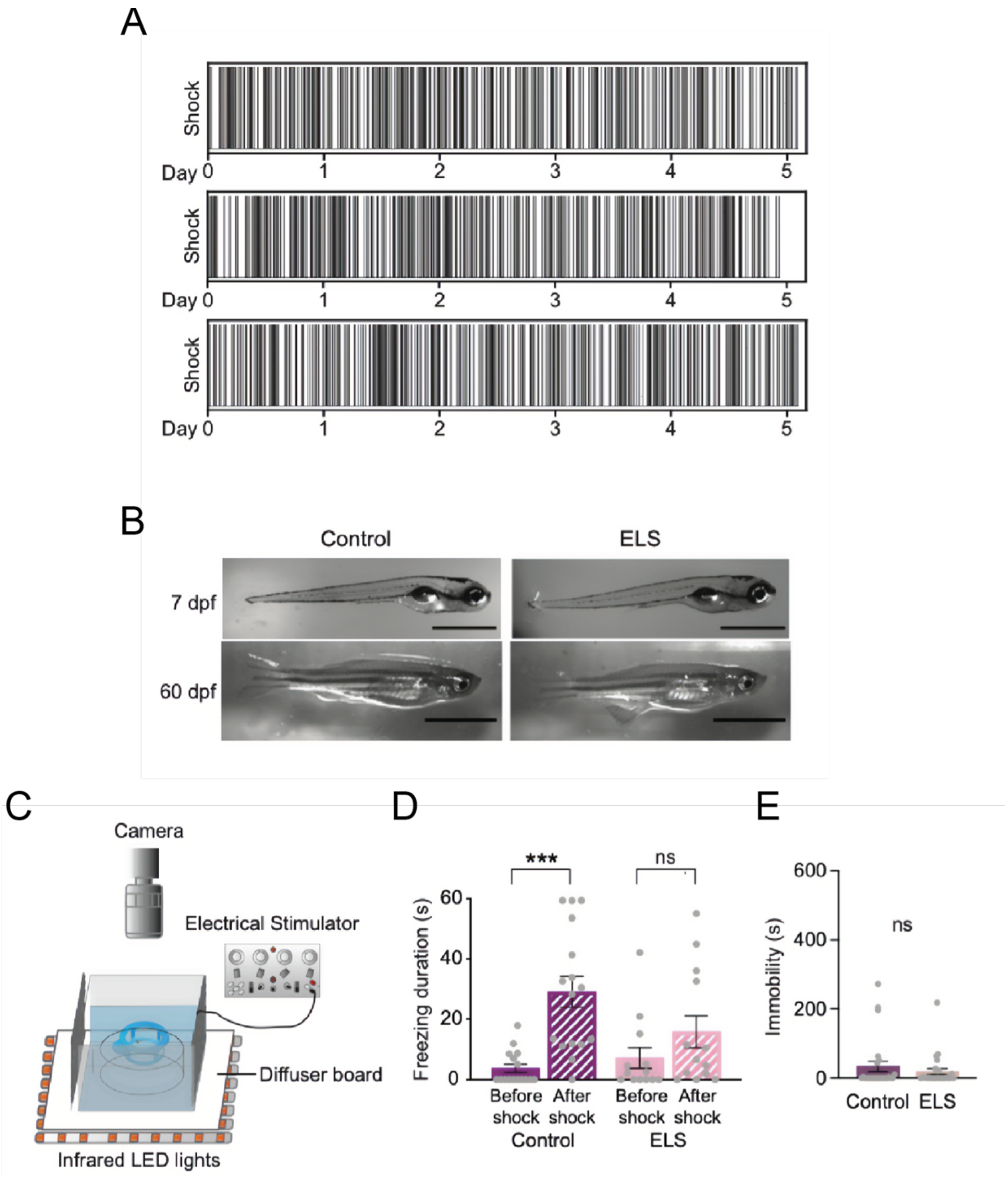

Figure S1. Mild electric shocks in ELS paradigm are random and cause large movements in larvae upon stimulation. (A) Representative graphs of random mild electric shocks applied throughout the five-day setup using a custom written script. Each line in the graphs represent an instance of shock applied. (B) Images of control and ELS siblings at 7 and 60 dpf revealed no gross physical defects. Scale bars in 7 and 60 dpf images represent 1mm and 5mm, respectively. (c) Diagram of shock assay performed on 7dpf larvae, a day after the ELS paradigm. Locomotor behavior was recorded a minute before shock stimuli and a minute immediately after the stimuli. (D) 7 dpf ELS larvae showed dampened 'freezing' response to electric shock compared to control larvae.

Statistical analysis was done using the Kruskal Wallis test followed by Dunn's multiple comparisons post-hoc test. Controls: n= 16; ELS: n= 13. Control before shock vs. Control after shock: p=0.0002; ELS before shock vs. ELS after shock: p= 0.84; Control before shock vs. ELS before shock: p> 0.99; Control after shock vs. ELS after shock: p= 0.19. (E) Quantification of total duration of immobility in the novel tank test at 60 dpf showed no difference between ELS (n= 27) and control (n= 25) siblings (Unpaired t test, p= 0.36).

Error bars show ± standard error of the mean. Asterisks denote statistical significance (***: p= 0.0005). ns denotes no significance.
