# Supplementary material for: Long lasting anxiety following early life stress is dependent on glucocorticoid signaling in zebrafish
Source: Sci Rep. 2022 Jul 27;12:12826. doi: 10.1038/s41598-022-16257-5 (PMC9329305; doi:10.1038/s41598-022-16257-5)
Supplement: Supplementary file 2 — Supplementary Figure S2. [file 41598_2022_16257_MOESM2_ESM.pdf]

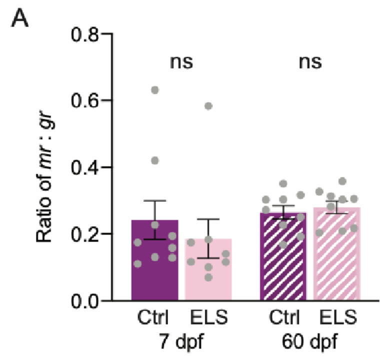

Figure S2. Analysis of MR: GR ratio at 7 and 60 dpf revealed no differences between controls and ELS animals. Statistical analysis was done using the Mann-Whitney test. 7dpf - Control: n= 9; ELS: n= 8, p= 0.20. 60 dpf – Control: n= 9; ELS: n= 9, p= 0.49. ns denotes no significance.
