# Supplementary material for: Long lasting anxiety following early life stress is dependent on glucocorticoid signaling in zebrafish
Source: Sci Rep. 2022 Jul 27;12:12826. doi: 10.1038/s41598-022-16257-5 (PMC9329305; doi:10.1038/s41598-022-16257-5)
Supplement: Supplementary file 3 — Supplementary Figure S3. [file 41598_2022_16257_MOESM3_ESM.pdf]

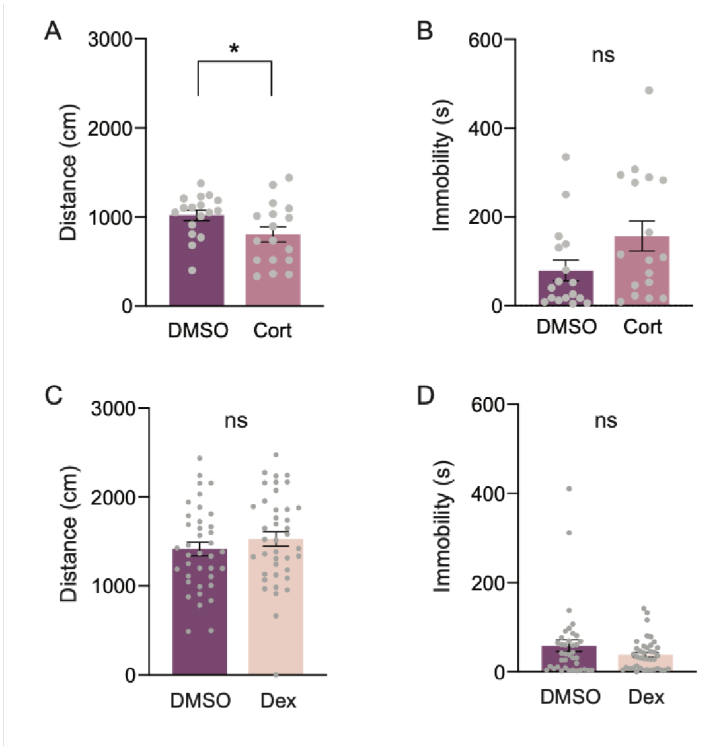

Figure S3. Analysis of locomotor behavior of drug-treated animals. (A) Total distance traveled was significantly reduced in Cort individuals compared to control DMSO siblings (Unpaired t test, p= 0.045). N= 17 per group. (B) Total durations spent immobile were no different between Cort and DMSO siblings (Unpaired t test, p= 0.068). N= 17 per group. (C) Analysis of distance traveled in the novel tank revealed no significant differences between control DMSO (n= 38) and Dex (n= 40) animals (Unpaired t test, p= 0.32). (D) Similarly, control DMSO (n= 38) and Dex (n= 40) siblings spent comparable amounts of time being immobile (Unpaired t test, p= 0.16).

Asterisks denote statistical significance (*: p= 0.05). ns denotes no significance.
