# Supplementary material for: Long lasting anxiety following early life stress is dependent on glucocorticoid signaling in zebrafish
Source: Sci Rep. 2022 Jul 27;12:12826. doi: 10.1038/s41598-022-16257-5 (PMC9329305; doi:10.1038/s41598-022-16257-5)
Supplement: Supplementary file 4 — Supplementary Figure S4. [file 41598_2022_16257_MOESM4_ESM.pdf]

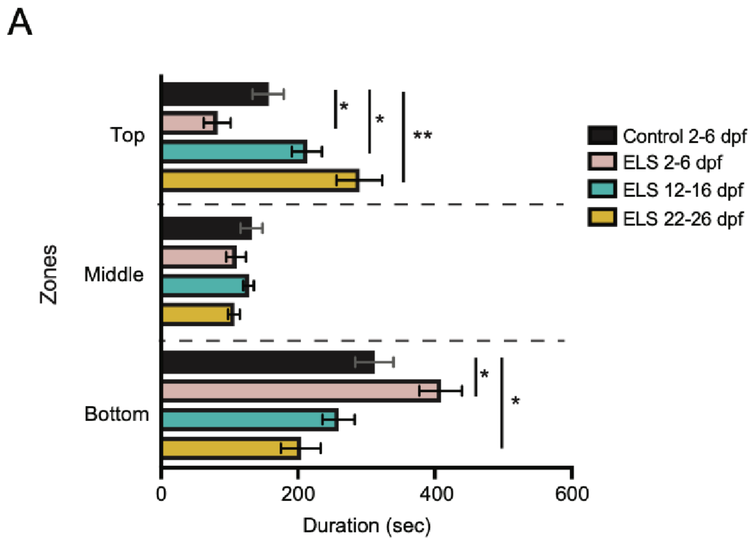

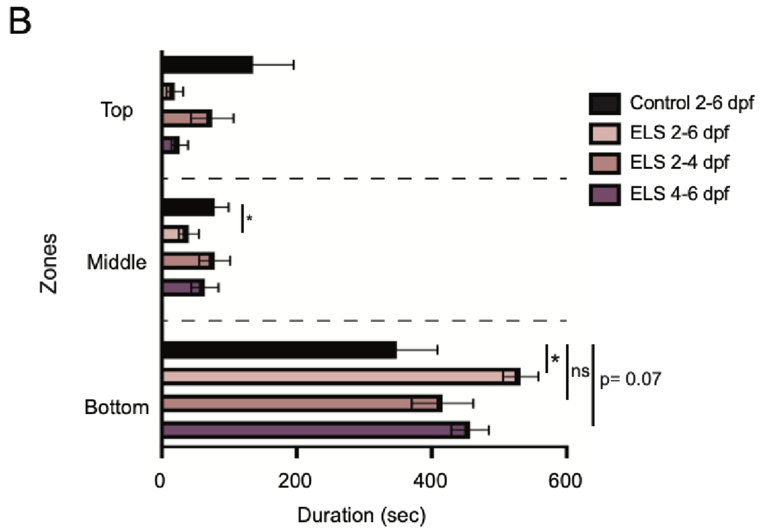

Figure S4. Quantification of durations in all zones in a novel tank test. (A) Quantification of durations in all zones revealed that only chronic stress in early life impacts anxiety behavior late in life. Multiple unpaired t-tests were performed between control and ELS siblings at three time windows. Top: Control vs. ELS 2-6 dpf: p=0.0098; Control vs. ELS 12-16 dpf: p= 0.041; Control vs. ELS 22-26 dpf: p= 0.0015; Middle: Control vs. ELS 2-6 dpf : p= 0.014; Control vs. ELS 12-16 dpf: p= 0.40; Control vs. ELS 22-26 dpf : p= 0.078; Bottom: Control vs. ELS 2-6 dpf : p= 0.014; Control dpf vs. ELS 12-16 dpf: p= 0.081; Control dpf vs. ELS 22-26 dpf : p= 0.081. Control: n= 15; ELS 2-6 dpf: n= 15; ELS 12-16 dpf: n= 16; ELS 22-26 dpf: n= 16. (B) Quantification of durations spent in the three zones in the novel tank suggested that stress between 4-6 dpf may be sufficient to cause increased bottomdwelling behavior later in life. Multiple unpaired t-tests were used to compare between groups in each zone. Top zone- Control vs. ELS 2-6 dpf: p=0.041; Control vs. ELS 2-4 dpf: p= 0.37; Control vs. ELS 4-6 dpf: p= 0.065. Middle zone- Control vs. ELS 2-6 dpf: p=0.0056; Control vs. ELS 2-4 dpf: p= 0.43; Control vs. ELS 4-6

dpf: p= 0.13. Bottom zone- Control vs. ELS 2-6 dpf: p=0.0026; Control vs. ELS 2-4 dpf: p= 0.31; Control vs. ELS 4-6 dpf: p= 0.07. Controls: n= 11; ELS 2-6 dpf: n= 10; ELS 2-4 dpf: n= 12; ELS 4-6 dpf: n= 11.

Error bars show ± standard error of the mean. Asterisks denote statistical significance (*: p= 0.05, **: p= 0.005). ns denotes no significance.
