# Supplementary material for: Long lasting anxiety following early life stress is dependent on glucocorticoid signaling in zebrafish
Source: Sci Rep. 2022 Jul 27;12:12826. doi: 10.1038/s41598-022-16257-5 (PMC9329305; doi:10.1038/s41598-022-16257-5)
Supplement: Supplementary file 5 — Supplementary Table S1. [file 41598_2022_16257_MOESM5_ESM.pdf]

Primers	Sequence (5' -> 3')
Crhb forward	TTC CAC CGC CGT ATG AAT GT
Crhb reverse	GGG GAG AAG TCG GGT TTC TG
Nr3c1 (gr) forward	GGC CAG TTT ATG CTT TTC CA
Nr3c1 (gr) reverse	CTT CCG CAA GTG AGA ACT CC
Nr3c2 (mr) forward	TAA TTC ACC CGC ATC TCC TC
Nr3c2 (mr) reverse	GAG CCA GGA GTT TGT TCG TC
Tuba1c forward	CCC AGG GCT GTC TTT GTA GA
Tuba1c reverse	GGT GGA ACA GCT GAC GGT AT
Actb1 forward	GCT CTC TTC CAG CCT TCC TT
Actb1 reverse	GCA CTG TGT TGG CAT ACA GG

Supplementary Table 1. List of RT-qPCT primers used in this study.
